# Editorial: Perioperative optimization of patients undergoing pancreatic surgery

**DOI:** 10.3389/fonc.2023.1170409

**Published:** 2023-03-03

**Authors:** Jorg Kleeff, Johannes Klose, Artur Rebelo, Ulrich Ronellenfitsch

**Affiliations:** Department of Abdominal, Vascular and Endocrine Surgery, Martin-Luther-University Halle-Wittenberg, University Medical Center Halle (Saale), Halle (Saale), Germany

**Keywords:** pancreatic surgery, pancreatic surgery complications, pancreatic surgery outcomes, pancreatic cancer, perioperative care

Pancreatic surgery has gained in volume in many parts of the world in recent years. This is mainly caused by the rising incidence of pancreatic cancer, for which resection is the only potentially curative treatment modality. There are also indications for pancreatic surgery in specific scenarios of benign diseases such as chronic pancreatitis. For example, the number of pancreatic resections carried out in Germany in 2021 was about 12,000, which equals a rate of roughly 15 per 100,000 inhabitants (1). Notwithstanding this rise in volume, pancreatic resections still bear a relevant risk of complications and death. While the benchmark for mortality is assumed 2% for pancreatic head resection and 1% for distal pancreatectomy, the benchmark for morbidity is between 50% and 60% for both surgeries (2). Yet, in broad clinical practice, these targets are not always reached, with mortality depending on a variety of factors such as hospital and surgeon volume (3). Thus, it is evident that optimal selection, preparation and intra- and postoperative treatment of patients is required to increase the likelihood of a favorable postoperative outcome.

The appropriate indication for surgery in patients with pancreatic cancer is paramount. Interdisciplinary tumor boards play an important role in weighing expected benefits against risks of the available treatments and can recommend a treatment on an evidence basis. Even if technical resectability is given, patients with a high risk of subsequent tumor recurrence and thus poor oncological prognosis will most likely not benefit from upfront surgery. While as of now there is no unanimously accepted modality to predict recurrence risk and survival, a number of potential parameters deserve consideration and further validation. In this special issue, Yang et al. have demonstrated that high serum concentrations of Gremlin 1 (GREM1), a regulator of bone morphogenetic protein signaling, predict shorter survival. This makes it a promising candidate to be potentially used as an adjunct to carbohydrate antigen 19-9 (Ca 19-9), which so far is the only biomarker routinely employed to evaluate the prognosis of patients with pancreatic cancer, but is not without limitations in its applicability (4). In fact, Ca 19-9 serum levels are affected by cholestasis. Wu et al.’s study suggests that Ca 19-9 levels should be adjusted for total bilirubin levels and clinical stage to enhance their prognostic value. Nomograms are a good instrument to assess the prognosis based on several factors. Guo et al. have developed and validated such a nomogram incorporating age, tumor size, leukocyte count, lymphocyte/monocyte ratio and albumin for predicting lymph node metastases. While local lymph node metastases do not pose a contraindication to resection, they indicate more advanced disease and might influence the decision for or against neoadjuvant therapy. Finally, the study by Cheng et al. suggests that the level of folate receptor-positive circulating tumor cells could predict recurrence and survival in patients with pancreatic adenocarcinoma.

Most patients with cancers located in the pancreatic head or periampullary region are jaundiced upon diagnosis. There is an ongoing debate if preoperative biliary drainage should be aimed for, with improved hepatic function as an argument in favor and the risk of interventional complications and infection of the pancreato-biliary duct system as arguments against it (5). Pattarapuntakul et al. show in their series of patients undergoing pancreatic head resections for periampullary lesions, the majority of which were pancreatic adenocarcinoma, ampullary adenocarcinoma, and cholangiocarcinoma, that preoperative bile drainage was not associated with 1-year survival, but with a lower risk of intraoperative bleeding and bile leakage. Based on their analyses, they recommend drainage for severely jaundiced patients, suggesting a threshold of 14.6 mg/dL.

Intraoperative techniques play an important role with regard to postoperative morbidity. While in pancreatoduodenectomy, the pancreatic anastomosis is a crucial element with postoperative pancreatic fistula (POPF) being one of the most impacting complications (6), in distal pancreatectomy the closure of the resection margin is of relevance to avoid POPF. In 2011, the multi-centre randomized DISPACT trial failed to show superiority of stapler versus suture closure of the remnant (7). Consequently, there is no uniform standard for margin closure. In a propensity-matched analysis, Tian et al. show a considerably lower incidence of POPF for lockstitch-enforced staple line closure compared with staple line closure alone. This result requires verification in randomized trials. Drain placement and timing of removal remain issues of controversy in pancreatic surgery and particularly so in pancreatoduodenectomy. In line with the results of recent meta-analyses on the topic, Xie et al. show in a propensity-matched analysis that drain removal on postoperative day three is safe.

After resection of pancreatic cancer and successful recovery of the patient, estimating the risk of recurrence is important to decide about the expected benefit from generally recommended adjuvant treatment and to guide follow-up. Tong et al. developed and validated a nomogram for predicting the risk of subsequent hepatic metastases incorporating postoperative Ca-125 level, tumor differentiation and size, lymph node ratio and venous invasion.

In summary, pre- intra- and postoperative management of patients undergoing pancreatic resections is crucial to achieve the best outcomes both in terms of postoperative morbidity and mortality and in terms of oncological outcomes for patients with cancer. The studies contained in this special issue have suggested some promising approaches, which require and deserve further validation in prospective studies.

## Author contributions

All authors have drafted the manuscript, provided intellectual content, and approved of the final version of the manuscript.
